# A Translational Study on Acute Traumatic Brain Injury: High Incidence of Epileptiform Activity on Human and Rat Electrocorticograms and Histological Correlates in Rats

**DOI:** 10.3390/brainsci10090570

**Published:** 2020-08-19

**Authors:** Ilia G. Komoltsev, Mikhail V. Sinkin, Aleksandra A. Volkova, Elizaveta A. Smirnova, Margarita R. Novikova, Olga O. Kordonskaya, Alexander E. Talypov, Alla B. Guekht, Vladimir V. Krylov, Natalia V. Gulyaeva

**Affiliations:** 1 Laboratory of Functional Biochemistry of the Nervous System, Institute of Higher Nervous Activity and Neurophysiology, Russian Academy of Sciences, 5A Butlerov Str., 117485 Moscow, Russia; komoltsev.ilia@gmail.com (I.G.K.); aleksandra.al.volkova@gmail.com (A.A.V.); liz0nka@mail.ru (E.A.S.); mrnovikova.ihna@mail.ru (M.R.N.); 2 Research and Clinical Center for Neuropsychiatry of Moscow Healthcare Department, 43 Donskaya Str., 115419 Moscow, Russia; guekht@mail.ru; 3 Sklifosovsky Research Institute of Emergency Care, 3 Bolshaya Sucharevskaya Square, 129010 Moscow, Russia; mvsinkin@gmail.com (M.V.S.); dr.kochetkova@mail.ru (O.O.K.); dr.talypov@mail.ru (A.E.T.); manuscript@inbox.ru (V.V.K.); 4 Department of Neurosurgery and Neuroresuscitation, Moscow State University of Medicine and Dentistry, 20/1 Delegatskaya Str., 127473 Moscow, Russia

**Keywords:** traumatic brain injury, post-traumatic epilepsy, epileptiform discharges, electrocorticograms, local field potentials, neocortex, hippocampus, microglia, neurodegeneration

## Abstract

Background: In humans, early pathological activity on invasive electrocorticograms (ECoGs) and its putative association with pathomorphology in the early period of traumatic brain injury (TBI) remains obscure. Methods: We assessed pathological activity on scalp electroencephalograms (EEGs) and ECoGs in patients with acute TBI, early electrophysiological changes after lateral fluid percussion brain injury (FPI), and electrophysiological correlates of hippocampal damage (microgliosis and neuronal loss), a week after TBI in rats. Results: Epileptiform activity on ECoGs was evident in 86% of patients during the acute period of TBI, ECoGs being more sensitive to epileptiform and periodic discharges. A “brush-like” ECoG pattern superimposed over rhythmic delta activity and periodic discharge was described for the first time in acute TBI. In rats, FPI increased high-amplitude spike incidence in the neocortex and, most expressed, in the ipsilateral hippocampus, induced hippocampal microgliosis and neuronal loss, ipsilateral dentate gyrus being most vulnerable, a week after TBI. Epileptiform spike incidence correlated with microglial cell density and neuronal loss in the ipsilateral hippocampus. Conclusion: Epileptiform activity is frequent in the acute period of TBI period and is associated with distant hippocampal damage on a microscopic level. This damage is probably involved in late consequences of TBI. The FPI model is suitable for exploring pathogenetic mechanisms of post-traumatic disorders.

## 1. Introduction

Traumatic brain injury (TBI) is an immense problem affecting predominantly young people [1]. The disease is associated with both high mortality in severe TBI and long-lasting consequences including cognitive disturbances, emotional disorders, and post-traumatic epilepsy [2]. Electrophysiological evaluation of brain activity in the acute period of TBI is helpful for revealing functional brain damage and identifying acute complications (nonconvulsive status epilepticus (NCSE), cycling electrographic seizures (ES), etc.). These harmful conditions need to be treated as soon as possible since they result in severe and often irreversible brain damage [3].

Human studies in this field have essential limitations. First, the sensitivity of scalp electroencephalograms (EEGs) can be lower than that of electrocorticograms (ECoGs) [4], limiting our understanding of pathological activity in the acute period of TBI in humans. Second, there are no direct noninvasive methods routinely available to reveal damage on mesoscopic and microscopic levels (level of cells) in humans, potentially resulting in an underestimation of the consequences of early epileptic discharge (ED). Third, the long period of epileptogenesis and development of other comorbidities complicate clinical studies and contributing to possibly overlooking the relationship between acute damage and late seizures. Therefore, the occurrence of epileptiform discharges (EDs) in the acute period of TBI on human ECoGs may remain covert, and their consequences obscure. Since the effects of EDs appearing in a nonepileptic brain (e.g., after acute insults) are unknown, no clear recommendations exist regarding epileptiform discharges (EDs) not accompanied by seizures or NCSE.

Translational electrophysiological studies have contributed to establishing connections among clinical and experimental researches. Lateral fluid percussion (FPI) brain injury in rats is one of most widely used TBI models leading to late post-traumatic seizures, epileptiform activity, cognitive disturbances, as well as hippocampal damage [5]. However, univocal links between early hippocampal damage and early epileptiform activity in rats have not been demonstrated.

This study consists of two parts, a clinical part and an experimental part. We characterized epileptiform activity, periodic patterns and electrographic seizures in patients with acute TBI on scalp EEGs and ECoGs. To study mechanisms of early epileptiform activity, we assessed early electrophysiological changes after FPI in rats and its validity for a translational study. Finally, we evaluated electrophysiological correlates of hippocampal damage, as well as behavioral changes a week after TBI in rats.

## 2. Materials and Methods

**Figure 1:**
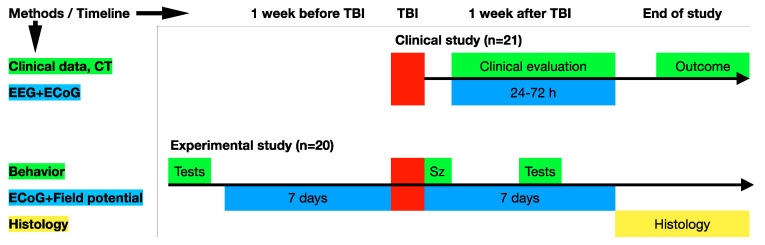
Study design flowchart. The study includes clinical and experimental parts. Direct translational links between them are provided through clinical and behavioral data concerning the severity of trauma and electrical activity recorded from the surface of the cerebral cortex (electrocorticograms (ECoGs)). Sz, immediate seizures.

The flowchart of the experimental design is presented in Figure 1. Obviously, clinical studies have more limitations in the design; important background information and the earliest seizures may not be well documented. In addition, morphological consequences of TBI on a microscopic level cannot be obtained. Translational links between clinical and experimental parts in this study included clinical and behavioral information about the severity of trauma and electrical activity on the surface of the cerebral cortex (ECoGs). The animal study also included background data, immediate post-traumatic seizures, and a histological analysis.

### 2.1. The Human Study

All clinical study procedures were conducted in accordance with the applicable laws and guidelines, good clinical practice, and ethical standards, and were approved by the local Ethic commission of the Sklifosovsky Research Institute of Emergency Care.

#### 2.1.1. Group Description

Twenty-one patients with acute TBI admitted to the Sklifosovsky Research Institute of Emergency Care and subjected to surgical treatment with a decompressive craniotomy in 2017–2020 were included in this study (mostly isolated TBI, possible combination with limb fractures, and uncomplicated rib fractures). Twenty male and 1 female patient aged 18 to 60 years (average 33.7 years) had a Glasgow Coma Scale (GCS) score at admission from 4 to 15 (average 10.6). Loss of consciousness, up to 30 min, was identified in 8 patients, and more than 24 h in 10 patients. Focal neurological deficits were revealed in 16 patients. Alcohol intoxication and drunkenness on admission were revealed in 4 patients. The lesion type on a head computed tomography (CT) scan included the following: intracerebral contusion (all volumes and localization); subarachnoid, intraventricular, intraparenchymal, acute subdural, or epidural hemorrhage (all volumes and localization); depressed skull fracture with dural penetration; extent of midline shift (more than 5 mm); or cisternal compression. Linear skull fractures without dural penetration were detected in 12 patients and depressed skull fractures with dural penetration in 4 patients. Intracerebral contusion was revealed in 13 patients (volume 17 ± 4 mm^3^), subarachnoid hemorrhage (SAH) in 15 patients, epidural hematoma (EDH) in 6 patients (volume 80 ± 26 mm^3^), subdural hematoma (SDH) in 19 patients (volume 61 ± 12 mm^3^), and intraventricular hemorrhage in 1 patient. Midline shift at first CT was 8.1 ± 1.5 mm. Diffuse axonal injury (DAI) was diagnosed in 3 patients.

The following clinical data were collected: mechanism and energy of trauma, GCS; co-morbidities; CT (the 1st and on the day after electrode implantation); type of surgical intervention, i.e., craniotomy or decompressive craniectomy; complications of TBI (liquorrhea, pneumonia, multiple organ dysfunction syndrome (MODS), meningitis, meningoencephalitis, sepsis, etc.); Glasgow Outcome Scale (GOS) at discharge and outcomes.

#### 2.1.2. Exclusion Criteria

Exclusion criteria included the following: accompanying malignant diseases; severe combined trauma (fractures of bones of a pelvis, bones of hips, the complicated fractures of ribs, damage to internal organs of abdomen and chest cavities); acute bleeding (supine tachycardia >100/min, increased respiratory rate >30/min, supine or postural hypotension); hypoxia at a prehospital stage (low level, less than 20 mm Hg) of saturation at the time of admission, massive ischemic brain injury on CT; pregnancy; previous TBI with severe old lesion on CT/MRI, with a history of loss of consciousness (less than 10 points of GCS); clinical inborn brain lesion causing disability; patients with known epilepsy before TBI.

#### 2.1.3. Electroencephalograms (EEGs) and Electrocorticograms (ECoGs)

An ECoG was recorded in patients subjected to surgical treatment with a decompressive craniotomy. Median interval after admission and first ECoG was 1 day (lower and upper quartiles, 0 and 2 days, respectively). One or two subdural strips of 4 or 6 platinum contact electrodes (Ad-Tech Medical Instruments Corporation IS04R-SP10X-000, TS06R-AP10X-0W6, Oak Creek, USA) were implanted on the craniotomy side for 24–72 h with subsequent CT control (additional CT after electrodes implantation). Electrodes were placed on frontal convexity (19% of patients), parietal convexity (5%), temporal convexity (61%), or basal part of temporal lobe (5%). Long term EEG monitoring after ECoG electrode implantation was performed using up to 12 ECoG strip electrodes, + 8 scalp EEG electrodes, + corkscrew reference and ground scalp electrodes. Recordings were performed on Natus Xltec (Canada), Compumedics, Mitsar (Russia) with a sampling rate of 500–2000 Hz. A total 763 h of EEGs (37 ± 5 h per patient) were analyzed in this study.

Scalp EEG (sEEG) and ECoG evaluation included detection of epileptiform discharges (ED), including focal spikes (S), spike-wave discharges (SW), sharp waves (Sh) or sharp wave-slow wave complex (ShW), polyspikes or sharp waves (PS, PSW) or runs of S and ShW, as well as diffuse or focal slowing. Rhythmic and periodic patterns defined using the standardized American Clinical Neurophysiology Society (ACNS) terminology (rhythmic delta activity (RDA), periodic discharge (PD)) were also evaluated. Additionally, we investigated and classified stable combinations of graphoelements. We described a new EcoG pattern, looking like “brushes”, superimposed with RDA and PD. Brushes were defined as visible fast activity (over 15 Hz) on RDA or PD on invasive recordings. We further analyzed features of this stable combination of pathological activity and the respective literature in the Discussion section. The prevalence and incidence of sporadic and continuous graphoelements was described according to ACNS terminology [6].

The sEEG and ECoG sensitivity was compared with respect to ED, RDA, and PD detection. For that purpose, we defined the discordance between sEEG and ECoG as the presence of ED, RDA, and PD only on sEEGs or ECoGs, or differences in the ED index (for example, rare on sEEGs vs. frequent on ECoGs). Different morphology of EDs or rhythmic patterns was not considered to be discordance (for example, SW on sEEGs vs. S on ECoGs, and PD on sEEGs vs. PD with brushes on ECoGs).

In addition, evaluation of the background activity (continuous EEG, presence of posterior dominant rhythm, or sleep graphoelements, etc.) and diagnosis of subclinical or nonconvulsive seizures defined according to Salzburg criteria were performed using sEEGs.

### 2.2. Animals

All procedures were performed in accordance with the ARRIVE guidelines and the UK Animals (Scientific Procedures) Act, 1986 and associated guidelines, EU Directive 2010/63/EU for animal experiments, or the National Institutes of Health guide for the care and use of Laboratory animals (NIH Publications no. 8023, revised 1978). The Ethical Commission of the Institute of Higher Nervous Activity and Neurophysiology of RAS approved our protocol.

The experiments were performed on 20 adult male Sprague Dawley rats (from the “Stolbovaya” Breeding Center, Moscow Region, Russia), body weight 449 ± 17 g (aged 8–10 months) at the beginning of the experiments. Exclusion criteria were unsuccessful surgery, damaged dura mater, presence of adhesive on dura mater, or inappropriate TBI induction. Two rats were excluded from the experiment due to unsuccessful surgical operation (1 during electrode implantation and 1 during craniotomy), one rat was excluded due to rapid weight loss (17%) and inactive behavior between the two operations. The other 17 rats were included into the experiment as sham-operated rats (*n* = 5) and rats with TBI (*n* = 12). During the experiment, the animals were housed in individual plastic cages at standard laboratory conditions (12 h light–dark cycle) with free access to food and water. All animals were handled during one week prior to the experiment.

#### 2.2.1. Electrode Implantation

For all surgical manipulations 1–3% isoflurane anesthesia was used. The animals were mounted in a stereotaxic frame, and four stainless steel screw epidural electrodes were implanted bilaterally over the frontal (AP 1 mm, ML ± 3 mm) and parietal (AP 7 mm, ML ± 3 mm) cortex; two depth nichrome electrodes were implanted in the hippocampal dentate gyrus (AP 6 mm, ML ± 4 mm, H 4.3 mm). The reference electrode was implanted in the occipital bone over the midline. The electrodes were fixed by dental cement and the area for the further craniotomy remained intact.

#### 2.2.2. TBI Induction

Lateral fluid percussion brain injury (FPI) was performed on day 7 after electrode implantation. The animals were anesthetized, and 4 mm diameter craniotomy was centered in the parietal bone (AP 3 mm, ML 3 mm). The integrity of the dura mater was inspected visually. A female luer-loc hub was attached to the craniotomy site with cyanoacrylate adhesive followed by dental cement. The animals were removed from the stereotaxic frame and, after recovery from anesthesia, connected to the FPI apparatus with an 80 cm polyvinylchloride tube (d = 3 mm). Fluid percussion of 2.7 ± 0.13 atm (corresponding to severe injury) was delivered to animals during free behavior. Seizures immediately after FPI were estimated visually. The sham-operated rats underwent all abovementioned procedures except for FPI.

#### 2.2.3. In Vivo Electrophysiology

The video, cortical electrocorticograms (ECoGs), and hippocampal local field potentials (HLFP) were synchronously recorded in home cages 24 h/day, for 7 days, after electrode implantation. Following 7 days of baseline recording video, ECoGs and HLFP registration 24 h/day was continued up to day 7 after FPI (thus, total time of continuous recording was 14 days). ECoG and HLFP were recorded using a wireless 8 channels biopotential measurement system BR8 (Bio Recorder) with 500 Hz sampling rate. The movements were traced with an accelerometer. For 24/7 video registration, a BestDVR-805Light recorder was used.

After the end of the video ECoG and HLFP monitoring period, 24 h long records were selected from each animal on day 6 of baseline recording (a day before TBI induction) and on day 7 after craniotomy. Recordings were filtered in EDF browser with Butterworth band pass filter 1–30 Hz and divided into 20 s epochs. The epochs containing high-amplitude spikes were selected manually, numbers of epochs with spikes were counted for each rat and were divided by the time period of recording analyzed (spike/h). A high-amplitude spike (S) was defined as a brief deviation of potential followed by a brief return to baseline, with or without slow wave (SW), appearing at least in two channels and negative in at least one of the channels. The spikes accompanied by the synchronous changes on the accelerometer were excluded as possible artifacts. The scoring was blinded. A total of 598 h of rat EEGs were analyzed (24.5 h per animal before and 25.3 h per animal after the craniotomy). We identified 810 epochs with spikes (181 before and 629 after the craniotomy; 99 of the epochs were identified in sham-operated rats, and 711 of the epochs in the rats with TBI).

#### 2.2.4. Behavioral Testing

To assess depressive behavior, a forced swim test (Porsolt test) was performed 2 weeks before electrode implantation and 7 days after craniotomy. The rats were placed in the high transparent cylinder (65 cm high, 30 cm in diameter) filled with water (temperature about 23–24 °C) for the 300 s. The number and duration of active and passive swimmings (immobilization), as well as the number and duration of dives, were assessed.

Fear conditioning was used to access context memory after TBI. The chamber with two compartments (light and dark) with a slatted floor was used. Learning session was performed the day before electrode implantation. The rats were placed in the light compartment for 300 s, 3 times, with 20 min intervals between sessions to explore the chamber. During the third session, when the rat left the light compartment, the doors between the sections were closed and electroshock was delivered through the floor of the dark compartment. The testing of the context memory was performed twice, on day 6 after electrode implantation and day 6 after the craniotomy. The rats were placed in the light compartment for 300 s, the latency to enter the dark compartment was recorded.

To assess anxiety behavior, a light-dark box (LDB) test was performed 2 days before the electrode implantation and on day 5 after the craniotomy. The LDB chamber, height 27 cm, consisted of two compartments, i.e., light (27 × 27 cm) and dark (18 × 27 cm). The rats were placed in the light compartment of the LDB and the following parameters were measured during 300 sec: the latency to enter the dark box, the vertical activity, the number of lookings outside from the dark box.

#### 2.2.5. Histology and Immunohistochemistry

On day 7 after FPI, the animals were deeply anesthetized with chloral hydrate (395 mg/kg intraperitoneally) and subjected to a transcardial perfusion with 4% paraformaldehyde in phosphate buffer (pH 7.4). The brains were removed and post-fixed in the same solution. Frontal sections (50 μm, 6 sections for each animal) made 2.1–5.8 mm caudal to the bregma with a 600 μm interval were used for the morphological analysis.

A part of slices was stained using cresylviolet (Nissl staining), another part was stained immunohistochemically for the microglial marker Iba1. Floating slices were washed in PBS, and then rinsed in PBST (0.01-M PBS with 0.3% Triton X-100). The slices were incubated in a blocking solution (5% normal goat serum (MP Biomedicals, Irvine, CA, USA) in PBST), and then incubated overnight in the solution containing rabbit anti-Iba1 antibody (Wako, Neuss, Germany) at 4 °C. Then, the sections were thoroughly washed in PBST and incubated in the solution containing Alexa Fluor 488 goat antibody to rabbit IgG (1:500, Invitrogen, Carlsbad, CA, USA) for 2 h. Sections were mounted with ProLong Gold Antifade with DAPI (Invitrogen, Carlsbad, CA, USA) and coverslipped.

#### 2.2.6. Morphometry

For histological analysis, the microphotographs were made on Keyence BZ-X700. Neocortical damage was assessed semiquantitatively using Nissl and anti-Iba staining using the following scale: 1 point, mild changes with gliosis, thinning of several cortical layers, and subtle neuronal loss in this area; 2 points, moderate changes with prominent gliosis, marked neuronal loss and thinning of all cortical layers; 3 points, necrotic changes in the cortex with prominent gliosis and neuronal loss around the necrotic area. The density of microglial cells in 150 × 150 μm visual field on sections stained immunohistochemically was calculated for 5–6 slices per animal in the polymorph layer of the ipsilateral and contralateral hippocampal dentate gyrus (DG), CA3 and CA1 fields. Similarly, neuronal density was assessed using Nissl stained sections. All measurements were made using ImageJ program.

### 2.3. Statistical Analysis

Statistical analysis was performed using STATISTICA 12 software (StatSoft, Tulsa, OK, USA). Visualization and correlation analysis were made using Graph Prism 8. For human data analysis, Fischer exact test, two tailed, was used to compare sEEG vs. ECoG sensitivity or mortality predictors; the Mann–Whitney U-test was used to compare groups with and without brushes on ECoGs. ECoGs, HLFP, and morphological data differences among groups in the animal study (sham vs. TBI) were analyzed using the Mann–Whitney U-test. ECoG and HLFP data differences between baseline and day 7 after craniotomy, and differences in morphological data between the hemispheres were analyzed using the Wilcoxon test. For analyses of fear conditioning, ANOVA for repeated measures was used. All data are presented as mean ± SEM (standard error of mean).

## 3. Results

### 3.1. Clinical Study

Nine of 21 patients (42.9%) died during the hospitalization. We analyzed predictors of mortality, including TBI characteristics, EEG patterns, and CT data. There were no significant predictors found except for DAI (three patients with DAI died, *p* < 0.05, Fischer exact test). This result can be explained by the limited cohort of severe patients with medical indications for craniotomy (decompression, intracerebral hemorrhage).

Pathological activities in sEEGs and ECoGs for each patient are presented in the Table 1. We did not find significant relationships between the type or localization of the injury and the type of pathological activity. To compare sensitivity of sEEGs and ECoGs, we analyzed pathological activity separately for scalp and intracranial recordings.

**Figure 2:**
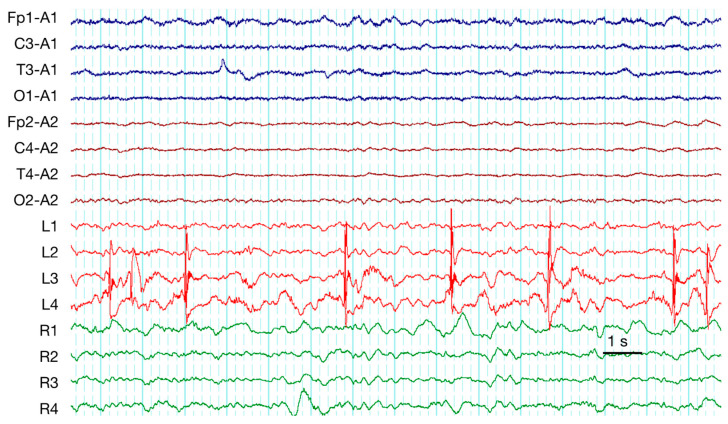
Interictal spikes in a patient 1 day after TBI. High-amplitude spikes are evident on ECoGs (L3–L4 electrodes on the strip) but not in scalp EEG. A typical example, patient #7.

**Figure 3:**
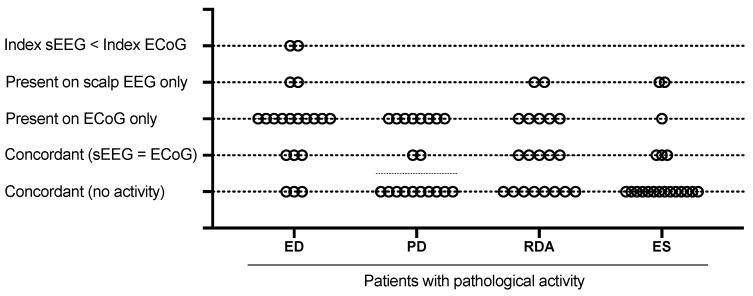
Distinctions in the pathological activities detected in scalp EEGs and ECoGs. The analysis of discordance between scalp EEG (sEEGs) and ECoGs was performed. Each dot denotes a patient with (or without) different pathological activity (left to right: ED, epileptiform discharge; RDA, rhythmic delta activity; PD, periodic discharges; ES, electrographic seizures). Concordance or discordance between sEEG and ECoG is shown on different lines. A discordance was identified if the activity was present either on sEEG or ECoG, or if differences in ED index changes were significant (for ED). The concordance was stated if the activity was presented or absent in both scalp and invasive electrodes. The ECoG was more sensitive with respect to ED and PD, and, in some patient, RDA and ES could be detected only with invasive electrodes, *n* = 21.

Epileptiform activity was detected on scalp EEGs of seven patients (35%) and on ECoGs of 16 patients (80%) (*p* < 0.05). Rare ED index was less common on ECoGs as compared with on sEEGs (4 of 16 vs. 1 of 6, *p* < 0.05). We detected a discordance between scalp and invasive recordings in 14 of 17 (82%) cases. In most cases (10 patients, 59%) ED was visible only on ECoGs (Figure 2), index of ED was higher on ECoGs of two patients (12%), and ED was visible only on sEEGs but not on ECoGs of two patients (12%). For ED, PD, RDA, and ES the discordance is presented graphically in Figure 3.

PD were noticed only on sEEGs of two (10%) patients, and on ECoGs of 10 patients (48%) (*p* < 0.05). In all cases with PD on sEEGs, they were also visible on ECoGs, the other eight cases (80%) were considered to be discordant. RDA was seen on sEEGs of seven patients (35%) and on ECoGs of 10 patients (48%). Discordance between sEEGs and ECoGs was observed in 7 of 12 cases with RDA (58%). RDA was only seen on ECoGs in five discordant cases (42%), and only on sEEG, but not on ECoGs, for two patients (17%).

Electrographic seizures were registered in seven patients; for four patients on sEEGs and for six patients on ECoGs. In three discordant cases, seizures were visible only on ECoGs of two patients and only on an sEEG for one patient. The difference in appearance of RDA and ES on sEEGs and ECoGs was not significant; in the case of ES, the nonsignificant difference was likely due to the small cohort of patients with electrographic seizures.

Nonconvulsive status epilepticus was diagnosed in five patients using sEEG (defined in five patients and possible in three patients, according to Salzburg criteria). Two of the patients with NCSE died during the hospitalization. ECoG was used only for the detection electrographic seizures. For NCSE diagnosis, ECoG was used only as an additional option, since there were no validated criteria for NCSE in invasive recordings. Since a lot of pathological activity was evident only on sEEGs, further investigations are needed to develop definite criteria of NCSE for invasive recordings.

**Figure 4:**
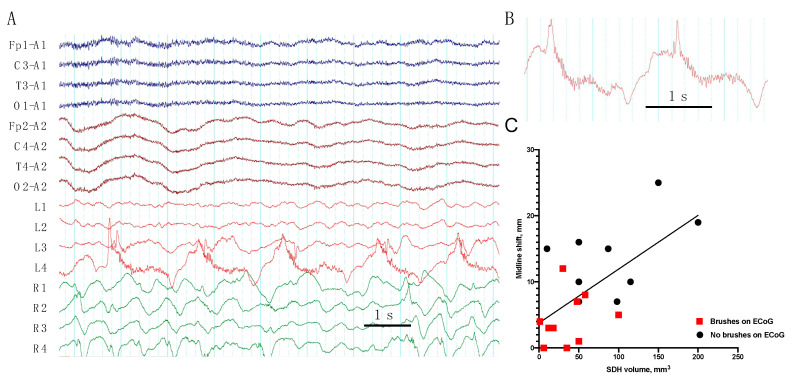
(**A**) Periodic discharges with the “brushes” on day 3 after TBI in patient #9. Periodic discharges with frequency 0.5 (complexes/sec) accompanied by fast (30–33 Hz) activity are evident on invasive ECoG (electrode L4); (**B**) The brushes from L4 electrodes; (**C**) Midline shift on CT correlates with subdural hematoma (SDH) volume (*p* < 0.005), the brushes appear in patients with low grade midline shift and SDH volume (*p* < 0.05, see text), *n* = 21.

The “brushes” were frequent on invasive recordings in acute TBI (11 patients, 52%), (Figure 4A,B); in 7 of 12 cases they were evident on the RDA and in 7 of 10 patients on the PD. The brushes were associated with epileptiform activity; in 9 of 11 (82%) patients with the brushes (on the RDA or PD), epileptiform discharges were presented in the same recordings. In addition, the brushes were seen on ECoGs in all three cases with modifiers on sEEGs (RDA + F or RDA + S). However, eight patients with ED had no brushes in the recordings. The presence of the brushes did not predict mortality (*p* = 0.67). While midline shift positively correlated with the volume of SDH (r = 0.39, *p* = 0.004), the brushes predominantly appeared in the patients with low grade midline shift (4.2 ± 1.1 mm vs. 12.4 ± 2.2 mm, *p* < 0.01) and lower SDH volume (35.8 ± 9.4 mm^3^ vs. 90.0 ± 19.6 mm^3^, *p* < 0.05), (Figure 4C).

Thus, among the patients with pathological activity, the brushes were seen only on ECoGs (and not on sEEGs) in 67% of patients with ED, in 77% of patients with PD, in 41% of patients with RDA, and in 29% of patients with ES. In general, ECoGs added new clinically relevant information about ED in 12 patients (60%), about PD in eight patients (40%), about RDA in five cases (25%), and about ES in two patients (10%). The detection of epileptiform abnormalities is important to choose the optimal treatment strategy (sedation, AEDs medication, etc.). A scalp EEG does not provide enough sensitivity for detection of epileptiform abnormalities and additional ECoG recordings for patients undergoing surgery is very helpful.

### 3.2. Experimental Study

The mortality after FPI was 41.7% (5 of 12 rats). The severity of trauma (pressure during FPI, atm) did not differ significantly between animals that died and survived (*p* = 0.18). Acute seizures were detected in all rats that died and in six of seven animals that survived (*p* = 1.0, Fischer exact test); four rats displayed clonic and seven rates displayed tonic-clonic seizures.

#### 3.2.1. Electrophysiological Analysis in Rats

**Figure 5:**
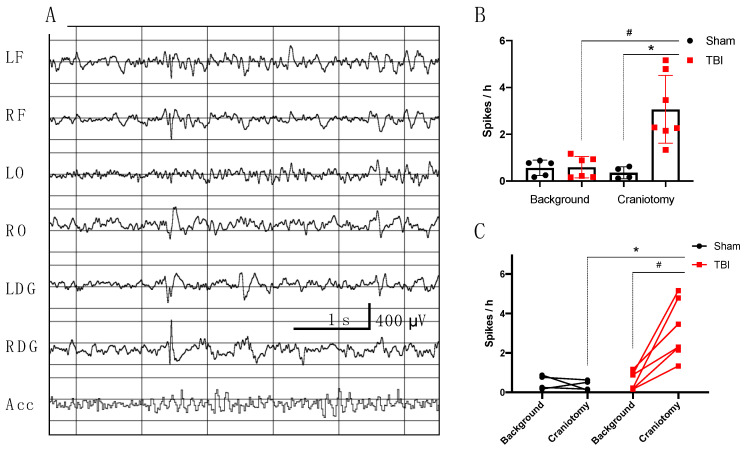
High-amplitude spikes before and after TBI in rats. (**A**) High-amplitude spikes 7 days after TBI. The spikes are evident on ECoG (LF, RF), but maximum amplitude was recorded in the ipsilateral hippocampal DG (HLFP); (**B**) Spike occurrence increased after TBI as compared with either background or sham-operated animals; (**C**) Spike occurrence in individual animals before and after TBI. *n* = 7 (TBI), n = 5 (Sham). * *p* < 0.05, Sham vs. TBI, Mann–Whitney test; # *p* < 0.05, background vs. craniotomy, Wilcoxon test; LF, left frontal cortex; RF, right frontal cortex; LO, left occipital cortex; RO, right occipital cortex; LDG, left dorsal hippocampus; RDG, right dorsal hippocampus; Acc, accelerometer.

During the first 24 h after FPI, rhythmic delta activity was evident in the ipsilateral hemisphere, resolving during the first day. We analyzed day seven after trauma as the most representative in the context of acute epileptiform changes. The number of epochs with high-amplitude epileptiform spikes (Figure 5A) in the hippocampus on the seventh day after FPI was 3.1 ± 1.4 epoch/hour, which was higher as compared with the background (0.6 ± 0.5 epochs/hour, *p* < 0.05) or shams (0.4 ± 0.3 epoch/hour, *p* < 0.01) (Figure 5B,C). Maximum amplitude of spikes was recorded in the ipsilateral hippocampus, accompanied by lower amplitude discharges in the contralateral hippocampus and in the cortex.

#### 3.2.2. Morphological Analysis

We assessed the damage to the cortex using Nissl and anti-Iba staining. Two rats with TBI had mild changes, three had moderate changes, and two had severe changes with necrosis in the cortex (2 ± 0.3 point). All rats had distinct microglial activation in the damaged area.

**Figure 6:**
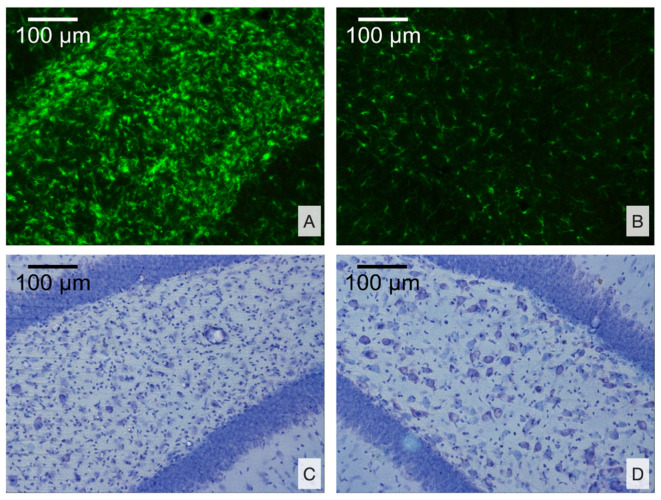
Histological alterations in the hippocampus 7 days after TBI. (**A**) Microglial activation in the dentate gyrus (DG) as compared with the contralateral hemisphere; (**B**) Iba-staining; (**C**) Loss of neurons in the polymorph layer of the DG as compared with the contralateral hemisphere; (**D**) Nissl staining.

**Figure 7:**
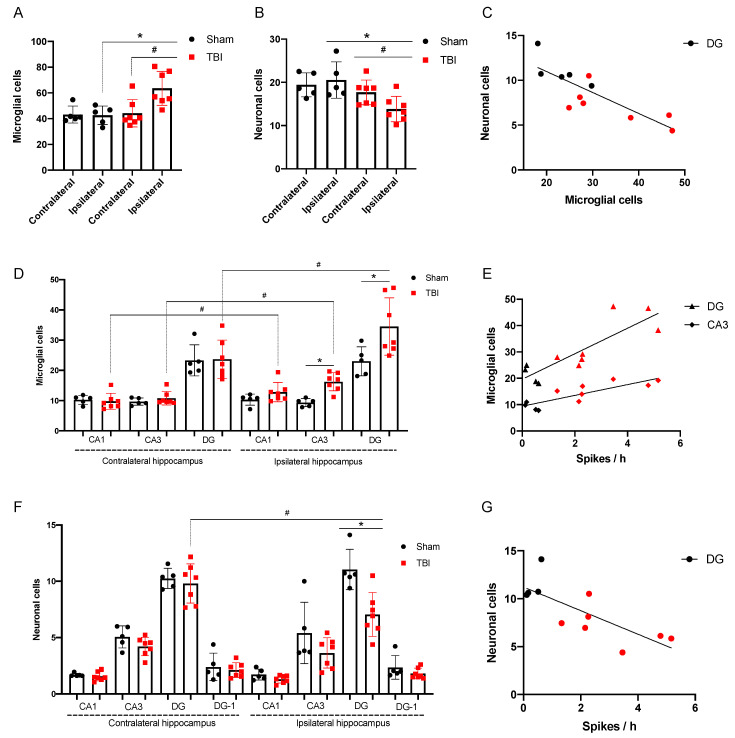
Distant damage to the hippocampus and spike occurrence. (**A**) Microglial cell density in the hippocampus (Iba-staining); (**B**) Neuronal cell density in the hippocampus (Nissl staining); (**C**) Neuronal cell loss correlates with microglial cell density in the ipsilateral dentate gyrus (DG) (red dots, rats with TBI and black dots, sham-operated animals, *p* = 0.001); (**D**) Microglial cell density in the hippocampal CA1 and CA3 fields and the DG. (**E**) Correlation between microglial cell density and spike occurrence on day 7 after TBI (*p* = 0.001); (**F**) Neuronal cell density in the hippocampal CA1 and CA3 fields, and the DG; (**G**) Correlation between neuronal cell density and spike occurrence on day 7 after TBI (*p* = 0.009). *n* = 7 (TBI) and *n* = 5 (Sham). * *p* < 0.05, Sham vs. TBI, Mann–Whitney test; # *p* < 0.05, ipsilateral vs. contralateral hemisphere, Wilcoxon test.

We focused on morphological changes in the hippocampus as the key structure most vulnerable to epileptic activity. Microglial cell density was higher and neuronal cell density was lower in the ipsilateral hippocampus of rats after TBI as compared with the contralateral hemisphere or sham-operated animals (*p* < 0.05, Figure 6 and Figure 7A,B). We analyzed these changes separately for the CA1 and CA3 fields, as well as the DG.

Microglial cell density in the ipsilateral CA3 and DG (polymorph layer) was 16.2 ± 3.0 and 34.5 ± 3.6 cells/field, respectively. It was higher as compared with either sham group (9.7 ± 1.2 and 23.0 ± 2.1 cells/field, respectively, *p* < 0.05) and as compared with the contralateral hemisphere (10.8 ± 2.2 and 23.7 ± 2.4 cells/field, respectively, *p* < 0.05). There were no changes in microglial density in the hippocampal CA1 field, in rats after TBI, as compared with shams, but microglial cell density was higher in the ipsilateral DG as compared with the contralateral hemisphere (12.8 ± 3.2 vs. 9.8 ± 2.6 cells/field, *p* < 0.05) (Figure 7D).

In the ipsilateral DG (polymorph layer), the number of neurons was 7.0 ± 1.9 cells/field and it was significantly lower as compared with the sham group (11.0 ± 1.8 cells/field, *p* < 0.05) and as compared with the contralateral hemisphere (9.8 ± 1.7 cells/field, *p* < 0.05). There were no changes in neuronal density detected in the radial layer of CA1 and CA3 fields of the hippocampus seven days after FPI (Figure 7F). A significant correlation was shown between the number of neurons and the number of microglia cells in the DG; in the rats with more pronounced activation of microglia, the number of neurons was lower (r = −0.82, *p* = 0.001) (Figure 7C).

#### 3.2.3. Spike Occurrence and Hippocampal Damage

The severity of histological changes significantly correlated with the number of spikes on the seventh day after FPI. In the rats with more numerous spikes, the number of neurons in the DG was lower (r = −0.76, *p* = 0.009) and the number of microglial cells was higher (r = 0.85, *p* = 0.001) (Figure 7E,G). Microglial cell density in the CA3 also correlated with spike occurrence (r = 0.84, *p* = 0.001) (Figure 7E).

#### 3.2.4. Behavior

In the forced swim test, FPI and sham-operated rats did not differ significantly in the number or duration of passive and active swimming, as well as in the latency to first immobility. In the LDB test, sham and FPI animals did not differ in the latency to enter the dark box, vertical activity, or the number of lookings outside from the dark box. In the fear conditioning test, none of the animals after sham operation entered the dark compartment where they previously had been exposed to the aversive stimulus (Figure S1). Thus, no behavioral differences could be revealed between experimental TBI and sham groups.

## 4. Discussion

### 4.1. Electrophysiological Characteristics of Acute TBI in Humans

In this study, we revealed a high incidence of epileptiform activity in TBI patients subjected to surgical treatment (86% of patients). According to our data, invasive ECoG was much more sensitive towards pathological activity, especially for epileptiform discharges and periodic patterns. Our results are consistent with studies on patients with epilepsy reporting ample intracranial activity that was not evident in the scalp recordings. A 10 cm^2^ of synchronous or temporally overlapping cortical activity is usually necessary to produce scalp-recordable EEG spikes [4]. A similar result was reported for ictal patterns; about 8–15 cm^2^ of cortex was necessary for its detection on EEGs [7]. However, so far, there have been no studies performed in acute insults such as TBI to directly compare the sensitivity of scalp and invasive EEGs for different acute EEG patterns (RDA, PD, etc.).

It has been previously reported that continuous EEG monitoring in patients with TBI revealed a high incidence of epileptic seizures (33%) and paroxysmal fast activity (26%) [8]. In our study, epileptiform abnormalities were even more frequent (86% of patients), and scalp EEG detected epileptiform activity in only 38% of cases, PD in 20% of cases, and in 57% of patients with ES as compared with scalp + invasive electrodes. Our result could partially explain the controversy in the prediction of post-traumatic epilepsy (PTE) by scalp EEG abnormalities [9] since most of abnormalities cannot be detected on scalp EEGs. In a large study (722 patients), EEGs failed to predict PTE [10]. The proportion of patients with and without EEG abnormalities was different only two years after TBI, possibly reflecting PTE rather than post-traumatic ED; among the patients developing PTE, in 49% of cases, at least one scalp EEG recording during the early period was normal. It was suggested that focal abnormalities on EEGs could serve as a risk factor for PTE only one month after injury [11], although another study showed that early sporadic epileptiform discharges on scalp EEG independently predicted post-traumatic epilepsy one year after TBI, as well as SDH [12].

Here, we described a new ECoG pattern which looked like “brushes”. On scalp EEGs, delta brushes are known as a hallmark of NMDA-encephalitis [13], but also it has been seen in other acute pathologies in intensive care units and in mesial temporal lobe epilepsy [14]. It has been shown that delta brushes represented a rare ictal pattern on invasive EEGs in patients with epilepsy [15,16]. Delta brushes are more often associated with high frequency oscillations, i.e., ripples and fast ripples [17]. In our study, brush pattern, superimposed with RDA and PD, was detected in 11 patients (52%) with acute brain injury. We found its association with epileptiform activity and with low grade midline shift and SDH volume. In addition, in our study, the brushes were seen in all patients with RDA plus modifiers. Modifiers on scalp EEGs are also mostly associated with seizures [18].

Many patients with high risks of PTE (for example, patients with SDH) have a clinical indication for craniotomy. Invasive EEG is a way to find a more confident biomarker of epileptogenesis and late post-traumatic pathology, including high frequency oscillations [19], possibly in combination with other pathological activity.

### 4.2. Translational Links between Clinical Study and Animal Model

All clinical studies in acute TBI patients have limitations. Usually, pre-TBI electrophysiological data is not accessible and the earliest seizures can be easily missed. Pathomorphological consequences of TBI on a microscopic level are not available in humans. Several new experimental approaches have been developed which are potentially relevant for studying changes on a mesoscopic level, for example, advanced MRI or optoacustic methods [20,21,22]. However, these approaches do not enable the performance of routine investigations on a cellular level for identifying morphological characteristics. We used ECoG as a translational link between the human study and experimental research. It is very important to find a mutual approach reflecting similar processes in animal and human brains. Electrical activity recorded from the surface of the cerebral cortex and without distorting the signal by skull and tissue with high spatial resolution (<5 mm^2^) may serve as such a link [23]. In both clinical and experimental studies, the incidence of epileptiform activity is very high, i.e., in 86% of patients and all rats with TBI. We cannot reliably determine sources of ED and different kinds of pathological activity on human ECoGs since the coverage of cortical surface is limited and no recordings from subcortical structures are available. However, in rats, high-amplitude hippocampal spikes are also visible on ECoGs, confirming a translational potential of our approach. In our study, more than 66% of patients had ECoG electrodes under temporal lobe, and it is highly possible that the detected ED also involved the hippocampus.

Importantly, mortality in both studies was about 40%, confirming the severity of TBI. Lateral fluid percussion brain injury model is widely used to study mechanisms of post-traumatic epilepsy [24,25]. The late period of TBI, in 20–40% of rats, is accompanied by seizures [5,26]; mesio-temporal seizures being predominant in the late period of TBI [27]. FPI has been used for multicenter research on predictors of post-traumatic epilepsy in animals [28]. Recently, we described elevation in the number of spike-wave discharge (SWD) occurrences in half of rats during the acute period after TBI, associated with freezing and anxiety-like behavior [29]. The experimental part of the present study includes data on the background state of animals (behavior, ECoG, and HLFP), immediate post-traumatic seizures, and histological analysis seven days after trauma, thus, filling limitations of the clinical study.

### 4.3. Distant Damage to the Hippocampus and Epileptiform Activity in Rats

We studied brain abnormalities related to post-traumatic epileptiform activity on a meso- and microscopic level, in rat LPI model inducing neocortical injury. It has been well documented that structural and functional changes in the hippocampus play a critical role in epileptogenesis [30,31]. Four weeks after pilocarpine-induced status epilepticus, the percentage of hilar ectopic granular cells in the dentate gyrus, mossy fiber sprouting, and mossy cell death were associated with seizure frequency and severity [32]. Loss of hilar neurons accompanied by increased granular cells excitability have been reported in the early and late period of TBI [33,34]. Specific vulnerable types of neurons in the dentate gyrus after TBI included different subtypes of GABA-ergic interneurons, and histological pattern of neuronal loss was similar to post-status epilepticus in rats [35]. We compared spontaneous occurrence of epileptiform spikes in the hippocampus with the severity of morphological changes a week after TBI. We showed, for the first time, that epileptiform spikes occurred even in the early period of the trauma, and their incidence was associated with significant neuronal loss and microglial activation in the hippocampal dentate gyrus. Thus, the development of hippocampal damage begins very early and is associated with epileptiform spikes.

**Figure 8:**
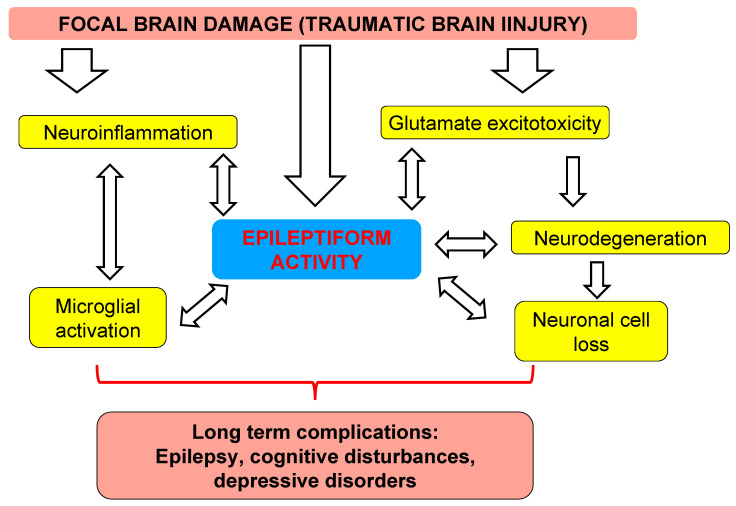
Putative interconnections among EDs and key mechanisms of remote hippocampal damage. Understanding the mechanisms of distant hippocampal damage is essential for the development of pathogenetically justified new treatment strategies to predict and prevent long-term consequences of TBI.

It is not clear whether structural damage to the hippocampus induced epileptiform abnormalities or, vice versa. Epileptiform activity is only a hallmark of neuroinflammation and progressive neuronal loss, but not its cause (Figure 8), which is a crucial question for understanding the mechanisms of PTE and its prevention. Distant damage to the hippocampus after TBI [35] shows similarity to damage induced by chemoconvulsants (kainate [36], dendrotoxin [37], and pentylenetetrazol [38,39]). Acute and excessive glutamate and aspartate releases lead to NMDA-receptor activation, Na+ and Ca2+ influx, and K+ efflux [40,41], inducing apoptotic and necrotic cell death as a result of excitotoxicity [42,43,44] and spreading of seizure activity [45,46]. Seizures also lead to progressive cognitive deficit [47] and cell proliferation in the dentate gyrus, although many clinical studies have shown that anti-seizure treatment affecting epileptiform activity in the early period of trauma failed to prevent late PTE [48,49]. The neuroinflammation is another pathophysiological link between focal damage and epileptiform activity. Neuroinflammation occurs in the acute post-traumatic period and induces edema and neurodegeneration [50]. It also takes place in the chronic phase of post-traumatic pathology and plays an important role in long-term complications after TBI [51]. Acute post-traumatic stress and corticosterone release is also an important link between bilateral neuroinflammation in the hippocampus and long-term consequences of trauma [52,53]. Cytokines produced by inflammatory cells (microglia and astrocytes) modify functioning of glutamate- and GABA-ergic receptors and voltage-gated ion channels, inhibit glutamate reuptake by astrocytes, provoke increased K+ extracellular concentration and, finally, may cause neuronal hypersynchronization and epileptiform activity [54]. The mechanisms of distant hippocampal damage need further investigations. In this study, we have shown that, in the early post-traumatic period, epileptiform activity and hippocampal damage are closely interrelated, although epileptiform activity may be a hallmark of secondary brain damage, but not necessarily its cause.

Cognitive disturbances were shown in rats after FPI from two weeks [55] and up to six months after TBI [56], and anxiety behavior one and three months after TBI [57]. We failed to demonstrate behavioral changes in the early post-traumatic period in rats, suggesting that despite prominent morphological abnormalities, no major emotional and cognitive alterations occurred, or the tests we used to reveal them were not sufficient. Most likely, immediate behavioral disturbances were compensated for, while stable post-traumatic emotional and cognitive disorders needed more time to become evident.

### 4.4. Clinical Implication

It is well known that SE treatment should be started as soon as possible, since prolonged epileptic activity continuing for more than 30 min can cause irreversible structural damage [3]. T2 = 30 min, currently used in practical definition of SE, was first reported in an animal model [58], then, it was included in a previous SE definition [59], and later the validity of T1 and T2 was proven in clinical practice. In our study, a relationship between early hippocampal damage and early epileptiform activity was demonstrated in rats, confirming pathophysiological links between functional and structural damage in acute TBI. Our study shows that epileptiform activity in the acute period of TBI can represent a potential biomarker of damage on a microscopic level, and this damage is possibly significantly involved in late consequences of TBI. More extensive and deep further investigations are needed for the development of treatment strategies in acute TBI to prevent its long-term complications.

## 5. Conclusions

1. ECoG is more sensitive for epileptiform activity and periodic patterns as compared with scalp EEG.
2. The incidence of epileptiform abnormalities is very high in patients subjected to surgical treatment after TBI.
3. We describe, for the first time, a “brush”-like pattern on RDA and PD on ECoG, associated with epileptiform activity, low grade midline shift, and subdural hematoma volume.
4. Lateral fluid percussion brain injury in rats is valid for studying early mechanisms of post-traumatic pathology.
5. Incidence of high amplitude epileptiform spikes is elevated seven days after FPI in rats with maximum amplitude in the ipsilateral hippocampus.
6. Microglial cell density is elevated in the CA3 field and DG of the ipsilateral hippocampus and is associated with neuronal loss in the polymorph layer of the ipsilateral DG.
7. The occurrence of epileptiform spikes correlates with microglial cell density and neuronal loss in the ipsilateral hippocampus of rat.

## Figures and Tables

**Table 1:** Pathological activities in scalp electroencephalograms (EEGs) and ECoGs, in patients after traumatic brain injury (TBI).

Patient	ED		PD		RDA		ES		NCSE	Injury (CT)
	sEEG	ECoG	sEEG	ECoG	sEEG	ECoG	sEEG	ECoG		
**1**		frequent SW		PD + brushes	RDA					EDH, SDH (Multiple)
2						RDA + brushes				EDH (Frontal), SDH (Temporal)
3										Contusion (Frontal), SDH (Multiple), SAH
4				PD + brushes		RDA + brushes				Contusion (Frontal), SDH (Multiple), SAH
5		abundant ShW								Contusion (Temporal), EDH, SDH (Multiple), SAH
6		occasion SW			RDA		cycl. Sz	cycl. Sz	Def	Contusion, EDH, SDH (Multiple), SAH
7		occasion SW (Figure 2)		PD + brushes	RDA + F	RDA + brushes			Pos	Contusion, EDH (Multiple), SAH
8	rare SW	rare S		PD		RDA				Contusion (Multiple), SAH
9		occasion SW		PD + brushes (Figure 3)	RDA	RDA + brushes	cycl. Sz	cycl. Sz	Def	Contusion, SDH (Multiple), SAH
10	rare Sh				RDA+S	RDA + brushes			Pos	Contusion (Frontal), SDH (Multiple), SAH
11	occasion SW	occasion S				RDA		Sz		SDH (Multiple), SAH
12	rare S	rare SW		PD + brushes						Contusion, EDH, SDH (Multiple)
13		frequent SW	PD	PD + brushes						SDH (Multiple), SAH
14		frequent runs SW			RDA	RDA + brushes	Sz	Sz		SDH (Multiple), SAH
15	rare SW	frequent runs S	PD	PD						SDH (Multiple)
16	rare SW						Sz		Def	DAI, SDH (Multiple), Ventricular hemorrage
17		abundant SW						cycl. Sz		Contusion (Temporal), SDH (Parietal), SAH
18	rare SW	frequent runs S								SDH (Parietal), SAH
19		frequent SW		PD + brushes		RDA				Contusion, DAI, SDH (Multiple), SAH
20		rare runs S			RDA + F	RDA + brushes				Contusion, DAI, SDH (Multiple), SAH
21	n/a	rare S	n/a	PD	n/a		n/a	Sz		Contusion, SDH (Multiple)
Proportion *	7/20 vs. 16/21 (*p* = 0.012)		2/20 vs. 10/21 (*p* = 0.015)		7/20 vs. 10/21 (*p* = 0.53)		4/20 vs. 6/21 (*p* = 0.48)			

sEEG, scalp electroencephalogram; ECoG, electrocorticogram; ED, epileptiform discharge; RDA, rhythmic delta activity (modifiers: F, fast activity and S, spikes), PD, periodic discharges; ES, electrographic seizures; NCSE, nonconvulsive status epilepticus (def, definite and pos, possible); S, focal spikes; SW, spike-wave discharge; Sh, sharp waves; ShW, sharp and slow wave complex; PS, polyspikes or sharp waves; EDH, epidural hematoma; SDH, subdural hematoma; SAH, subarachnoidal hematoma; DAI, diffuse axonal injury; Sz, seizures; Cycl. Sz, cyclic seizures. * proportion of patients with pathological activity on sEEGs or ECoGs within the cohort; Fischer exact test, two tailed.

## Supplementary Materials

The following are available online at https://www.mdpi.com/2076-3425/10/9/570/s1, Figure S1: Fear conditioning test in animals 7 days after TBI and respective Sham group.
