# Supplementary figures and images for: A Translational Study on Acute Traumatic Brain Injury: High Incidence of Epileptiform Activity on Human and Rat Electrocorticograms and Histological Correlates in Rats
Source: Brain Sci. 2020 Aug 19;10(9):570. doi: 10.3390/brainsci10090570 (PMC7565553; doi:10.3390/brainsci10090570)

### Fear Conditioning

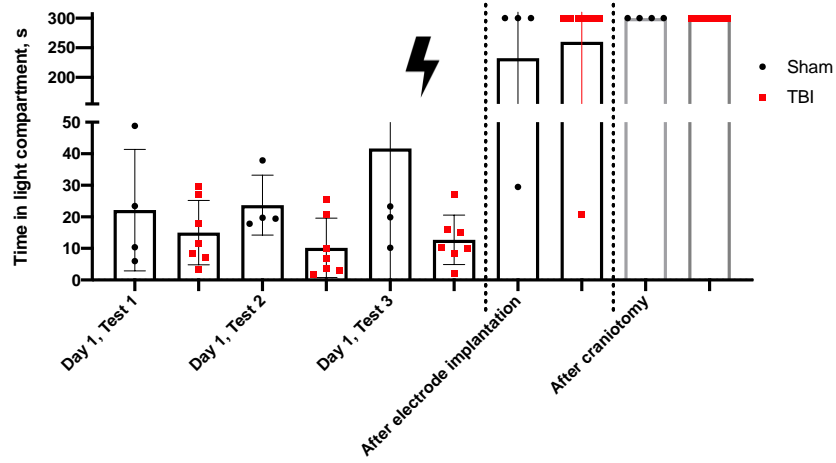

Supplement: Supplementary file 1 [file brainsci-10-00570-s001.pdf]
